# Supplementary material for: MetaRibo-Seq measures translation in microbiomes
Source: Nat Commun. 2020 Jun 29;11:3268. doi: 10.1038/s41467-020-17081-z (PMC7324362; doi:10.1038/s41467-020-17081-z)
Supplement: Supplementary file 10 — Supplementary Data 7 [file 41467_2020_17081_MOESM10_ESM.zip › File2/Confidence_High_Taxonomy/183545_out.krona.html]

Javascript must be enabled to view this page.

members
magnitude
magnitudeUnassigned
count
unassigned
taxon
rank

183545\_out

3

3
superkingdom
2

3
phylum
976

class
200643
3

171549
order
3

3
171552
family

3
838
genus

3
species

SRS017701\_contig\_number\_contig-100\_336.132119SRS053649\_contig\_number\_20963SRS145497\_contig\_number\_contig-100\_120.126093
165179
